# Plant-Mediated Synthesis of Silver Nanoparticles: Their Characteristic Properties and Therapeutic Applications

**DOI:** 10.1186/s11671-016-1257-4

**Published:** 2016-01-28

**Authors:** Ill-Min Chung, Inmyoung Park, Kim Seung-Hyun, Muthu Thiruvengadam, Govindasamy Rajakumar

**Affiliations:** Department of Applied Bioscience, College of Life and Environmental Science, Konkuk University, Seoul, 05029 South Korea; Department of Microbiology, Pusan National University, Busan, 609735 South Korea

**Keywords:** Green synthesis, Silver nanoparticles, Optimization, Characterization, Biomedical applications

## Abstract

Interest in “green nanotechnology” in nanoparticle biosynthesis is growing among researchers. Nanotechnologies, due to their physicochemical and biological properties, have applications in diverse fields, including drug delivery, sensors, optoelectronics, and magnetic devices. This review focuses on the green synthesis of silver nanoparticles (AgNPs) using plant sources. Green synthesis of nanoparticles is an eco-friendly approach, which should be further explored for the potential of different plants to synthesize nanoparticles. The sizes of AgNPs are in the range of 1 to 100 nm. Characterization of synthesized nanoparticles is accomplished through UV spectroscopy, X-ray diffraction, Fourier transform infrared spectroscopy, transmission electron microscopy, and scanning electron microscopy. AgNPs have great potential to act as antimicrobial agents. The green synthesis of AgNPs can be efficiently applied for future engineering and medical concerns. Different types of cancers can be treated and/or controlled by phytonanotechnology. The present review provides a comprehensive survey of plant-mediated synthesis of AgNPs with specific focus on their applications, e.g., antimicrobial, antioxidant, and anticancer activities.

## Review

### Introduction

**Fig. 1:**
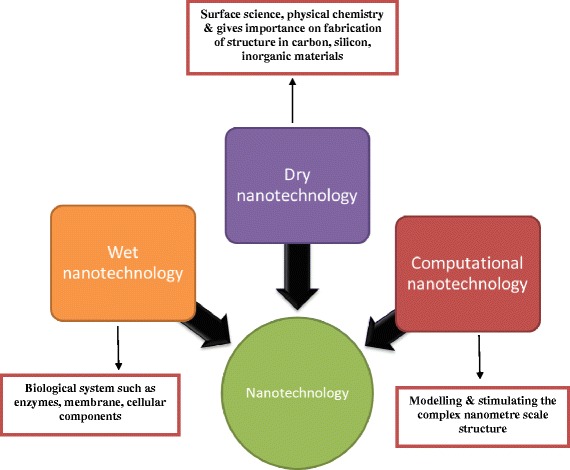
Different types of nanotechnology

The utilization of nanotechnology for constructing nanoscale products in research and development divisions is growing [1]. Nanotechnology can be used to produce a broad range of products applicable to an equally broad array of scientific sectors. “Creation,” “exploitation,” and “synthesis” are terms associated with nanotechnology, which generally considers materials that measure less than 1 mm. “Nano” is derived from the Greek word “nanos”, meaning “*dwarf*, *tiny*, or *very small*” [2]. Nanotechnologies are generally classified as *wet*, *dry*, and *computational. Wet nanotechnology* is associated with living organisms such as enzymes, tissues, membranes, and other cellular components. *Dry nanotechnology* is associated with physical chemistry and the production of inorganic items, such as silicon and carbon. *Computational nanotechnology* is associated with simulations of nanometer-sized structures [3]. These three dimensions (wet, dry, and computational) depend on each other for optimal functionality, represented in Fig. 1. Nanotechnology supports diverse unique industries, such as electronics, pesticides, medicine, and parasitology, and thus provides a platform for collaboration [4]. Nanobiotechnology provides one such example, wherein the study and development combine multiple scientific sectors, including nanotechnology, biotechnology, material science, physics, and chemistry [2, 5].

Biologically synthesized nanoparticles with antimicrobial, antioxidant, and anticancer properties are possible through the collaboration of different natural science sectors. These nanotechnologies may provide novel resources for the evaluation and development of newer, safer, and effective drug formulations [6].

### Different Modes of Nanoparticle Synthesis

**Fig. 2:**
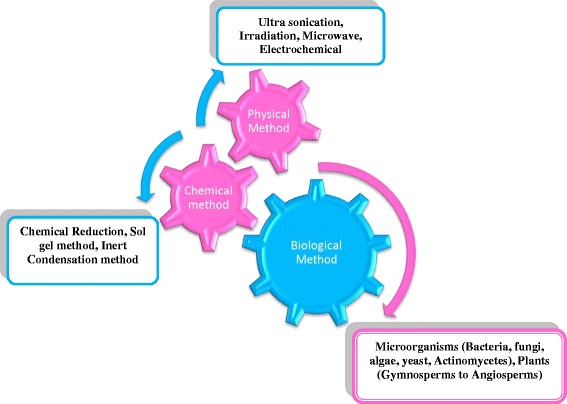
Methods involved in nanoparticle synthesis

Nanoparticles, which have unique properties due to their size, distribution, and morphology, are critical components of any nanotechnology. In the late 1970s, R.O. Becker et al. used silver particles to treat infections caused by microorganisms during the treatment of orthopedic diseases, resulting in faster bone recovery [7]. At present, varied physical, chemical, biological, and hybrid methods (Fig. 2) are utilized to synthesize distinct nanoparticles [8, 9]. The synthesis of nanoparticles has traditionally relied on two approaches, physical and chemical. These approaches include ion sputtering, solvothermal synthesis, reduction, and sol-gel techniques. Nanoparticle synthesis methods can also be classified as bottom-up and top-down. Chemical methods involve the reduction of chemicals [10], electrochemical procedures [11], and reduction of photochemicals [12]. Plant-based synthesis of nanoparticles is in contrast faster, safer and lighter; works at low temperatures; and requires only modest and environmentally safe components [13]. Plant-based nanoparticles have attracted more attention due to growing interest in environmentally conscious products. In addition, the synthesis of nanoparticles using plants offers other advantages, such as the utilization of safer solvents, decreased use of dangerous reagents, milder response conditions, feasibility, and their adaptability in use for medicinal, surgical, and pharmaceutical applications. [14]. Furthermore, physical requirements for their synthesis, including pressure, energy, temperature, and constituent materials, are trivial.

Nanoparticles made of noble metals have also received attention over the last few years, as they can be used in medicine, biology, material science, physics, and chemistry [15]. Among the several noble metal nanoparticles, silver nanoparticles (AgNPs) have attracted special attention due to their distinct properties, which include favorable electrical conductivity, chemical stability, and catalytic and antibacterial activity [12]. Silver at the nanoscale also has different properties from bulk silver. Synthesis of AgNPs is an emerging area and is much sought after [16]. The green synthesis of AgNPs has been accomplished using plants, microorganisms, and other biopolymers [12]. Wet chemical synthesis can be robustly scaled for the large-scale synthesis of AgNPs of tunable shape and size through optimization of synthesis conditions. However, wet chemical methods use toxic chemicals, which are hazardous for the environment and usually result in the adsorption of toxic chemicals on to the surface of synthesized AgNPs, making them unsuitable for biomedical applications. In contrast, physical methods are expensive and cumbersome for the large-scale production of nanoparticles. Therefore, the development of environmentally conscious, energy-efficient, facile, and rapid green synthesis methods that avoid toxic and hazardous chemicals has attracted significant interest [17]. In addition, due to their potent antimicrobial activity, AgNPs have also been used in clothing [18], foods [19], sunscreens, and cosmetics [20, 21].

### Synthesis of AgNPs

**Fig. 3:**
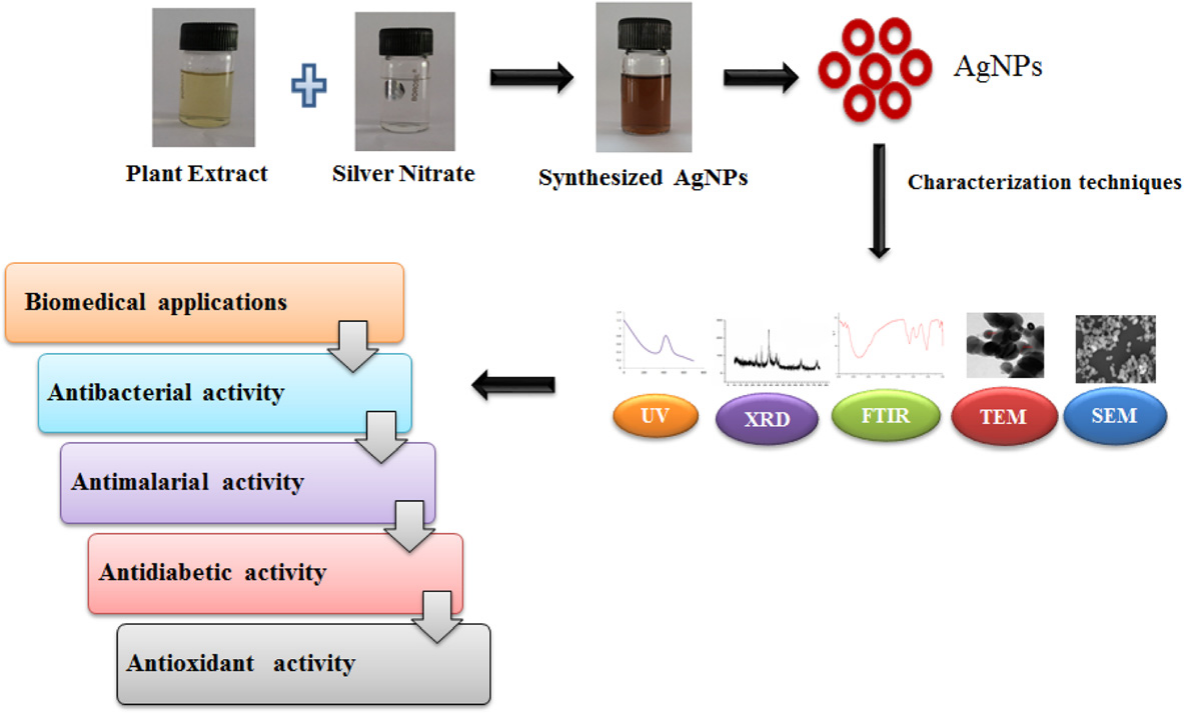
Plant-mediated synthesis of silver nanoparticles: their characteristic properties and therapeutic applications

The use of plants for nanoparticle synthesis offers a wide range of benefits over other biological synthesis methods because it does not require the maintenance of cell cultures and incorporates support for the large-scale synthesis of nanoparticles [22]. Extracellular nanoparticle synthesis, which utilizes extracts from individual leafs rather than entire plants, may prove to be more inexpensive due to easier downstream processing (Fig. 3). Sastry and his group are responsible for pioneering nanoparticle synthesis using plant extracts [22–27].

Green synthesis of AgNPs using plant extracts containing phytochemical agents has attracted considerable interest (Table 1). This environmentally friendly approach is more biocompatible and cost-efficient and includes the capability of supporting larger synthesis [28, 29]. The synthesis of AgNPs via different “green” chemico-physical conditions, as well as by numerous microorganisms, has been heavily investigated. When AgNPs are chemically synthesized, three main components are required: (1) silver salt (e.g., AgNO_3_), (2) a reducing agent (e.g., NaBH_4_), and (3) a stabilizing or capping agent (e.g., polyvinyl alcohol) for controlling the size of nanoparticles and preventing their aggregation [30]. AgNPs have applications in wound-healing, eye disease therapy, DNA processing, and pharmaceuticals in addition to other relevant mainstream applications: electronics, optics, catalysis, and Raman scattering [31–35]. Lokini et al. [36] showed that AgNPs could destabilize the outer membrane and rupture the plasma membrane, thereby depleting intracellular ATP. Silver has a greater affinity to react with sulfur or phosphorus-containing biomolecules in the cell; therefore, sulfur-containing proteins in the membrane or inside cells and phosphorus-containing elements like DNA are likely to be preferential sites for binding AgNPs. The advantages of using plants for the synthesis of nanoparticles include their availability, safety in handling, and presence of a variability of metabolites that may aid in reducing silver. The time required to reduce 90 % of silver ions is approximately 2 to 4 h [27]. Gericke and Pinches [37] reported that the size of particles that form intracellularly could be controlled by altering key factors such as pH, temperature, substrate concentration, and time of exposure to the substrate.

**Table 1:** Green synthesis of silver nanoparticles using different plant extracts

Plants	Plant parts	Size (nm)	Shape	References
*Prunus yedoensis*	Leaf	20–70	Circular, smooth edges	[84]
*Tephrosia tinctoria*	Stem	73	Spherical	[111]
*Grewia flaviscences*	Leaf	50–70	Spherical	[128]
*Skimmia laureola*	Leaf	46	Hexagonal	[81]
*Clerodendrum serratum*	Leaf	5–30	Spherical	[129]
*Averrhoa carambola*	Leaf	14	Spherical	[130]
*Rosmarinus officinalis*	Leaf	10–33	Spherical	[85]
*Carica papaya*	Leaf	50–250	Spherical	[131]
*Plukenetia volubilis*	Leaf	4–25	Optical	[132]
*Cucurbita maxima*	Petals	19	Crystalline	[97]
*Moringa oleifera*	Leaf	11	Rectangle	[97]
*Acorus calamus*	Rhizome	19	Spherical	[97]
*Aristolochia indica*	Leaf	30–55	Spherical or cubical	[133]
*Euphorbia helioscopia*	Leaf	2–14	Spherical	[134]
*Datura metel*	Leaf	40–60	Spherical	[135]
*Momordica cymbalaria*	Fruit	15.5	Spherical	[136]
*Hypnea musciformis*	Leaf	40–65	Spherical	[137]
*Potentilla fulgens*	Root	10–15	Spherical	[29]
*Annona muricata*	Leaf	20–53	Spherical	[138]
*Justicia adhatoda*	Leaf	5–50	Spherical	[139]
*Hemidesmus indicus*	Leaf	25.24	Spherical	[140]
*Emblica officinalis*	Leaf	15	Spherical	[141]
*Quercus brantii*	Leaf	6	Spherical and polydispersed	[142]
*Helicteres isora*	Root	30–40	Crystalline	[143]
*Saraca indica*	Leaf	23	Spherical	[144]
*Abutilon indicum*	Leaf	106	Crystalline	[145]
*Prosopis farcta*	Leaf	10.8	Spherical	[146]
*Mukia maderaspatana*	Leaf	13–34	Spherical	[147]
*Ficus carica*	Leaf	21	Crystalline	[148]
*Sinapis arvensis*	Seed	14	Spherical	[149]
*Ziziphus Jujuba*	Leaf	20–30	Crystalline	[65]
*Calotropis gigantea*	Latex	5–30	Spherical	[150]
*Nelumbo nucifera*	Root	16.7	Polydispersed	[151]
*Aerva lanata*	Leaf	18.62	Spherical	[152]
*Myrmecodia pendan*	Whole plant	10–20	Spherical	[153]
*Piper longum*	Fruit	46	Spherical	[57]
*Enteromorpha flexuosa*	Seaweed	2–32	Circular	[154]
*Lansium domesticum*	Fruit	10–30	Spherical	[155]
*Onosma dichroantha*	Root	5–65	Spherical	[86]
*Crataegus douglasii*	Fruit	29.28	Spherical	[156]
*Vitex negundo*	Leaf	≥20	Cubic	[69]
*Alstonia scholaris*	Bark	50	Spherical	[157]
*Lycopersicon esculentum*	Fruit	10–40	Spherical	[158]
*Musa balbisiana*	Leaf	50	Spherical	[159]
*Azadirachta indica*	Leaf	20	Triangular	[159]
*Ocimum tenuiflorum*	Leaf	50	Cuboidal	[159]
*Artocarpus heterophyllus*	Seed	10.78	Spherical and irregular	[71]
*Cocos nucifera*	Coir	22	Spherical	[160]
*Eucalyptus chapmaniana*	Leaf	60	Spherical	[52]
*Morinda citrifolia*	Root	30–55	Spherical	[161]
*Thuja occidentalis*	Whole plant	122	Spherical	[162]
*Hydrastis canadensis*	Whole plant	111	Spherical	[162]
*Phytolacca decandra*	Whole plant	90.87	Spherical	[162]

The biochemical and molecular mechanisms of AgNP biosynthesis remain poorly characterized and should be investigated to further optimize the process. For instance, characterization of biochemical mechanisms underscored the importance of phytochemicals, which may mediate biosynthesis. Improvements in chemical composition, size, shape, and dispersity of nanoparticles would permit the use of nanobiotechnology in a variety of other applications [38]. Plant crude extracts contain novel secondary metabolites such as phenolic acid, flavonoids, alkaloids, and terpenoids, which are mainly responsible for the reduction of ionic metal into bulk metallic nanoparticles [39]. Primary and secondary metabolites are constantly involved in redox reactions required to synthesize eco-friendly nanoparticles. Biosynthesis reactions can be modulated to transform the shape and size of nanoparticles by using different metal concentrations and amounts of plant extract in the reaction medium [27, 40].

*Capsicum annuum* leaf extracts contain a number of biomolecules, such as proteins, enzymes, polysaccharides, amino acids, and vitamins, which could act as bioreductants for metal ions or as scaffolds to direct the formation of AgNPs in solution. In detail, the mechanism underlying the bioreduction of silver was hypothesized to first involve trapping of silver ions on the surface of proteins in the extract via electrostatic interactions (i.e., recognition process). Silver ions are then reduced by proteins, leading to changes in their secondary structure and the formation of silver nuclei. Silver nuclei subsequently grow by the further reduction of silver ions and their accumulation at nuclei [41]. *Callicarpa maingayi* stem methanolic extracts were used for the synthesis of AgNPs, leading to the formation of [Ag (*Callicarpa maingayi*)]^+^ complex. Plant extracts contain aldehyde groups, which are responsible for the reduction of silver ions into metallic AgNPs. The different functional group, –C = 0, C = N, indicates amide I of polypeptides that are responsible for the capping of ionic substances into metallic nanoparticles. Molecular studies on the biosynthesis of silver crystals have revealed a complex process, which is not fully understood yet [42].

### Physical Requisites for the Synthesis of AgNPs

Easier, more reliable, and environmentally friendly methods to synthesize nanoparticles accelerate their widespread adoption, which would benefit humans and the environment [43]. Silver disassembles into particles following the addition of plant extract, which may lead to a color change. Solutions of AgNPs appear dark, yellow-brown in color because of the surface plasmon resonance phenomenon [44]. Gardea-Torresdey et al. [45] determined the influence of pH on the mass of nanoparticles when using alfalfa biomass in the biosynthesis of colloidal gold. Mock et al. [46] reached a similar conclusion that the unique pH conditions of different extracts affect nanoparticle size and shape. Two extracts from the same host plant may have a different pH, thus highlighting the need for better synthesis methods for nanoparticles. Large nanoparticles are most often formed only at lower pH values, instead of higher pH values, as has been previously reported [47, 48]. Dwivedi and Gopal [49], utilizing extracts of *Chenopodium album*, observed trivial variations in zeta potentials of nanoparticles in pH conditions ranging from 2 to 10 and determined that nanoparticles were more stable when exposed to higher pH conditions. Veerasamy et al. [50] demonstrated that mangosteen extracts induced the nucleation of a cluster of AgNPs at pH values over 4. Furthermore, nanoparticles grew rapidly, with their pH values ranging from basic to neutral. These results demonstrate the significant impact of pH on parameters of nanoparticles. The formation and growth of nanoparticles is retarded by acidic conditions, whereas basic conditions promote nanoparticle assembly. Larger nanoparticles are formed in lower pH conditions (pH 4), whereas significantly smaller nanoparticles are formed in higher pH conditions (pH 8). Our results indicate that the size of nanoparticles decreases when pH increases. pH values in the range of 2–14 play an important role in the synthesis of AgNPs. In plants, AgNP synthesis occurs at various pH values depending on the plant species [50]. However, previous studies have indicated that neutral pH is optimal for AgNP synthesis. At this pH, little or no assembly of AgNPs into particles of suitable size and shape occurs [51].

Newly synthesized AgNPs, formed within 60 min of incubation with leaf extracts of *Eucalyptus chapmaniana*, exhibited a UV-Vis peak at 413 nm [52]. The UV-Vis spectra of AgNPs, synthesized with leaf extracts of *Desmodium gangeticum* for an optimum incubation time of 90 min, exhibited a peak at 450 nm [53]. Vilchis-Nestor et al. [54] demonstrated a UV-Vis peak at 436 nm for AgNPs formed within 4 h of incubation with *Camellia sinensis* extracts. Chandran et al. [27] synthesized AgNPs with leaf extracts of *Aloe vera* incubated for 24 h, which exhibited a UV-Vis peak at 410 nm. UV-Vis absorption spectra reach a maximum when the synthesis of nanoparticles (NPs) is complete, which requires sufficient time for the nucleation and subsequent stabilization of nanoparticles. Song and colleagues synthesized stable AgNPs extracellularly, with average particle sizes ranging from 15 to 500 nm, with *Pinus densiflora*, *Diospyros kaki*, *Ginkgo biloba*, *Magnolia kobus*, and *Platanus orientalis* leaf extracts. The rate of synthesis and final conversion to AgNPs was faster with higher reaction temperatures. However, average particle sizes of nanoparticles produced with *D. kaki* leaf extracts decreased from 50 to 16 nm when the temperature of synthesis was increased from 25 to 95 °C [16]. *Ocimum sanctum* leaf extracts could reduce silver ions into crystalline AgNPs (4–30 nm) within 8 min of the reaction. These nanoparticles were likely stable due to the presence of proteins, which may act as capping agents. *O. sanctum* leaves contain ascorbic acid which was likely important for the reduction of silver ions into metallic AgNPs [55].

Monodisperse spherical AgNPs (~3 nm) were also synthesized using gum kondagogu (nontoxic polysaccharide derived as an exudate from the bark of *Cochlospermum gossypium*) [56].

### Characterization of Synthesized AgNPs

The synthesis of AgNPs using a 5:1 ratio of fruit extracts of *Piper longum* was evident by a change in the color of 1 mM AgNO_3_ solution from colorless to brownish-yellow, which resulted in a peak at 430 nm in UV-Vis spectra [57]. Aqueous leaf extracts of *Manilkara zapota* were used to synthesize AgNPs, which exhibited XRD with 2*θ* values of 38.06°, 44.37°, 64.51°, and 77.31° sets of lattice planes, which may be indexed to the (111), (200), (220), and (311) face-centered cubic (fcc) structure of silver, respectively [58].

Fourier transform infrared (FTIR) spectroscopy, which is used to evaluate chemical bonds in surface atoms and functional atoms on the surface of nanoparticles, can be used to characterize physical properties of nanomaterials and their functions [59, 60]. Certain proteins and metabolites, such as terpenoids or flavonoids that are present in leaf extracts of *Prosopis juliflora*, may be responsible for the decay and pause of AgNPs synthesis [61]. The FTIR spectra of AgNPs synthesized using either fresh or dried *Codium capitatum* extracts exhibited a strong transmission band at 1535 cm^−1^ corresponding to the bending vibration of secondary amines of proteins. The FTIR peak at 1637 cm^−1^ for AgNPs synthesized using *Andrographis paniculata* extracts can be attributed to the carbonyl stretch of amides and could be related to proteins that potentially cap AgNPs [62]. In *C. annuum* extracts, the formation of AgNPs is mediated by amine groups or the secondary structure of proteins [63].

The hydroxyl and carbonyl groups present in carbohydrates, flavonoids, terpenoids, and phenolic compounds are powerful reducing agents that may be responsible for the bioreduction of Ag^+^ ions necessary for AgNP synthesis. FTIR studies confirm that the carbonyl groups of amino acids and peptides of proteins have a strong affinity to bind metal ions, and they may encapsulate nanoparticles, forming a protective coat-like shell that prevents their further aggregation and leading to their stabilization in the medium [64].

A single-step method (biogenic) for the synthesis of AgNPs utilizes *Ziziphus jujuba* leaf extract as a reducing and stabilizing agent at room temperature. TEM images revealed nanoparticles featuring differing shapes and sizes, averaging 25 nm. These results were confirmed by DLS analysis, which revealed a hydrodynamic radius of 28 nm [65]. Environmentally friendly synthesis of AgNPs, which utilized *Argemone mexicana* leaf extracts and were 20 nm in size, had antimicrobial and antifungal activity against multiple bacterial and fungal pathogens [66]. Extracts from the Cycas leaf were utilized to prepare AgNPs measuring 2–6 nm [67]. AgNPs, measuring 14 nm and synthesized using *Solanum torvum* extracts, exhibited a peak at 434 nm in UV-Vis spectra. Using EDX analysis, Arunachalam et al. [28] showed that AgNPs were crystalline in nature and observed strong signal energy peaks for silver atoms in the range of 2–4 keV with weaker signals for carbon, oxygen, and chloride, which are prevalent biomolecules in *Memecylon umbellatum*. The size, shape, and size distribution of nanoparticles were observed by TEM and selected area electron diffraction (SAED) patterns of TEM images [68]. The crystalline nature of AgNPs was determined by SAED, which revealed fcc silver.

A facile biosynthesis method utilizing methanolic extracts of *Vitex negundo*, which can be performed at room temperature, was used to successfully synthesize spherical colloidal AgNPs of different sizes, although it required different reaction times. The sizes of colloidal AgNPs prepared for 6, 24, and 48 h averaged 10.11 ± 3.98, 12.80 ± 4.97, and 18.23 ± 8.85 nm, respectively [69]. The morphology and size of AgNPs synthesized using *Pulicaria glutinosa* extracts were examined by TEM, which revealed monodisperse spherical nanoparticles between 40 and 60 nm [70]. AgNPs synthesized using seed extracts of *Artocarpus heterophyllus* exhibited variance in their size, ranging from 3 to 25 nm with an average of 10.78 nm [71]. AgNPs fabricated using *Boerhaavia diffusa* leaf extracts as the nontoxic reducing agent and examined by TEM revealed AgNPs that were fcc structures of spherical shape and an average particle size of 25 nm [72].

### Biomedical Applications of AgNPs

#### Antimicrobial Activity of AgNPs

The threat posed by the potential outbreak of antibiotic-resistant microbes is growing globally and demands the introduction and production of novel more advanced platforms for the study and development of more potent antimicrobial agents against multidrug-resistant strains [73]. The antimicrobial activity of AgNPs is widely recognized, though their activity can change with physical characteristics of the nanoparticle, such as its shape, mass, size, and composition, and conditions of its synthesis, such as by pH, ions, and macromolecules [74]. Their shapes can be relevant to their antibacterial activity [75]. Compared to larger AgNPs, smaller AgNPs have a greater binding surface and show more bactericidal activity [76]. Variation in the thickness and molecular composition of the membrane structures of gram-positive and gram-negative bacteria account for the difference in their sensitivities to AgNPs [77]. Bactericidal activity is presumably due to changes in the structure of the bacterial cell wall as a result of interactions with embedded AgNPs, leading to increased membrane permeability and consequently death [78]. AgNPs also interact with sulfur- and phosphorus-rich biomaterials, which include intracellular components, such as proteins or DNA, and extracellular components such as membrane proteins. These components influence the respiration, division, and ultimately survival of cells [79]. Upon compromising the bacterial cell wall, silver ions (as part of AgNPs) can enter into cells, leading to the accumulation of damaged DNA and effect on protein synthesis [80].

AgNPs synthesized with *Skimmia laureola* leaf extracts have antibacterial activity, with maximum growth inhibition activity against *Staphylococcus aureus* (14.67 mM), followed by *Klebsiella pneumoniae*, *Pseudomonas aeruginosa* (14.33 mM), and *Escherichia coli* (11.67 mM) [81]. AgNPs synthesized with mangrove plant *Avicennia marina* extracts exhibited highest inhibition activity against *E. coli* (18.40 ± 0.97 mM) and lowest against *S. aureus* (10.87 ± 1.33 mM). Its minimum inhibitory concentration (MIC) and minimum bactericidal concentration (MBC) were 0.25 and 50.0 μg/mL, respectively, against select bacteria [82]. Sankar et al. [83] synthesized AgNPs with extracts of oregano (*Origanum vulgare*), which exhibited antimicrobial activity against human pathogens, including *Escherichia coli*, *Aeromonas hydrophila*, *Salmonella* spp., *Shigella dysenteriae*, *Salmonella paratyphi*, and *Shigella sonnei*. Furthermore, AgNPs were cytotoxic to human lung cancer lines (A549 cells), which killed 50 % of cells at 100 μg/mL. Sathishkumar et al. [84] evaluated the bactericidal activity of AgNPs synthesized with *Morinda citrifolia* leaf extracts against a wide range of human pathogens, such as *Escherichia coli*, *Pseudomonas aeruginosa*, *Klebsiella pneumoniae*, *Enterobacter aerogenes* (gram-negative), *Bacillus cereus*, and *Enterococcus* sp. (gram-positive). The antibacterial activity of AgNPs synthesized with *Rosmarinus officinalis* extracts was tested against gram-positive bacteria, and the maximum zones of inhibition at dosages of 20, 40, and 80 mg/disk were 21.52, 30, and 31.2 mm, respectively, against *S. aureus* and 13.4, 15.63, and 16.21 mm, respectively, against *Bacillus subtilis* [85]. The antimicrobial activity of the medicinal plant *Onosma dichroantha* and antimicrobial activity of silver chloride nanoparticles suggest a novel approach to the development of bactericides applicable to a wide range of applications, such as the treatment of burn wounds and injuries [86].

AgNPs synthesized with *Prunus yedoensis* leaf extracts exhibited significant antibacterial activity against two skin pathogens, *Propionibacterium acnes* and *Staphylococcus epidermidis*. Exhibited zone of inhibition (ZOI) sustained greater levels of AgNPs (30 μg) after 48 h of inhibition against two gram-positive bacteria; furthermore, tetracycline sulfate at volume of 100 μg/mL was evaluated [87]. AgNPs synthesized with *O. vulgare* leaf extracts have broad-spectrum antibacterial activity against nine different human pathogens. Greater than 10-mm zones of inhibition were observed against *Escherichia coli* (enteropathogenic, EP), *Aeromonas hydrophila*, *Salmonella paratyphi*, *Salmonella* sp., *Shigella dysenteriae*, and *Shigella sonnei*. This level of antibacterial activity was comparable to the standard antibiotic chloramphenicol [83]. Saxena et al. [88] synthesized AgNPs using *Ficus benghalensis* leaf extracts, and its bactericidal activity against *E. coli* was evaluated by the broth microdilution method. The bactericidal activity of AgNPs synthesized with *B. diffusa* plant extracts was evaluated against three fish bacterial pathogens, *Aeromonas hydrophila*, *Pseudomonas fluorescens*, and *Flavobacterium branchiophilum*. Of these, *F. branchiophilum* was more sensitive to AgNPs, and the other two pathogens were equally sensitive [72]. Tripathi et al. [89] evaluated bactericidal activity of silver nanoballs at a concentration of 40 μg/mL against *Escherichia coli*, *Salmonella typhimurium*, *Bacillus subtilis*, and *Pseudomonas aeruginosa* by measuring colony-forming units (CFU). Silver nanoballs prevented the growth of bacteria and induced toxicity.

AgNPs, which are filled with polyphenolic compounds, disrupt the cell walls of bacteria, which make gram-negative bacteria specifically sensitive. Polyphenolic compounds generate free radicals and other oxygen-based reactive species, which can induce considerable damage and toxicity [75]. Other damages may result as membranes become disrupted, including the widespread loss of K^+^ ions, leading to a decrease in membrane potential. Significant membrane disruption results in cytoplasmic leakage, which includes the discharge of proteins and lipopolysaccharide molecules. The outer membrane of bacteria is composed of lipopolysaccharides and is fundamentally asymmetric, while the inner membrane comprises tight chains of phospholipids, which are semi-permeable [75]. The exact mechanism of interaction between AgNPs and bacteria is not fully understood. AgNPs may attach to the cell wall and thus disrupt membrane permeability and ultimately cell respiration. AgNPs can also directly penetrate into cells since they may bind to cell wall proteins that contain sulfur and phosphorus-containing biomolecules such as DNA [90, 91]. Thus, they can easily bind to constituents of the bacterial cell and disturb normal functions of the cell. Another possible mechanism is the release of Ag cations, which are antibacterial, from AgNPs [92].

#### AgNPs in Cancer Control

AgNPs perform well as cancer therapeutics because they can disrupt the mitochondrial respiratory chain, which induces the generation of reactive oxygen species (ROS), and ATP synthesis, which can induce DNA damage [93, 94]. AgNPs synthesized with *Sesbania grandiflora* leaf extracts were demonstrated to be cytotoxic to MCF-7 cancer cells. Morphological characteristics, including the disruption of membrane integrity, decreased cell growth, cytoplasmic condensation, and cell clumping, were observed in MCF-7 cells treated with AgNPs, whereas control cells remained active. In addition, apoptotic features, such as cell shrinkage and nuclear condensation and fragmentation, were also observed in MCF-7 tumor cells 48 h after treatment with 20 μg/mL of AgNPs. AgNPs synthesized with *S. grandiflora* extracts induced the generation of free radicals, which resulted in oxidative damage and caspase-mediated apoptosis [95].

AgNPs synthesized with *Guignardia mangiferae* extracts exhibited potent antifungal activity against plant pathogenic fungi. IC_50_ values of AgNPs were 63.37, 27.54, and 23.84 μg/mL against normal African monkey kidney (Vero), HeLa (cervical), and MCF-7 (breast) cells, respectively, after a 24-h incubation period. Thus, AgNPs synthesized with *G. mangiferae* extracts are highly biocompatible, have potentially wider applicability, and should be explored as promising candidates for a variety of biomedical/pharmaceutical and agricultural applications [96]. AgNPs were synthesized using extracts from different plant origins: *Cucurbita maxima* (petals), *Moringa oleifera* (leaves), and *Acorus calamus* (rhizome). Among the three synthesized nanoparticles, AgNPs synthesized with *A. calamus* rhizome extracts had enhanced antimicrobial and anticancer activity, which were evaluated through MTT assays against epidermoid A431 carcinoma cells. AgNPs synthesized with *A. calamus* rhizome extracts were superior to AgNPs generated with petal and leaf extracts in their antimicrobial and anticancer activities [97]. Both treated (synthesized) and untreated AgNPs induced DNA fragmentation at all concentrations [98]. Compared to untreated cells, cells treated with AgNPs synthesized using *Phytolacca decandra*, *Hydrastis canadensis*, *Gelsemium sempervirens*, and *Thuja occidentalis* extracts exhibited DNA laddering, confirming the apoptotic effects of nanoparticles. Specifically, AgNPs synthesized using *P. decandra* and *G. sempervirens* extracts effectively induced DNA laddering compared to AgNPs synthesized using *H. canadensis* and *T. occidentalis* extracts [98].

The IC_50_ values of AgNPs synthesized using *Potentilla fulgens* extracts were 4.91 and 8.23 μg/mL in MCF-7 and U-87 cell lines, respectively. Furthermore, the cytotoxic effects of nanoparticles were evaluated against cancerous and normal cells using trypan blue assay and flow cytometric analysis. In contrast to their effect on normal cells, nanoparticles are capable of impairing or killing cancerous cells [29]. AgNPs synthesized with *Coleus amboinicus* extracts were cytotoxic to EAC cell lines. AgNPs induced 50 and 70 % cytotoxicity at 30 and 50 μg/mL, respectively, indicating concentration-dependent cytotoxicity [99]. AgNPs synthesized with alcoholic flower extracts of *Nyctanthes arbor-tristis* can be used for molecular imaging and drug delivery. Even at the highest concentration tested (250 μg/mL), AgNPs were only marginally toxic to L929 cells [100]. The anticancer activity of AgNPs synthesized with unripe fruits of *Solanum trilobatum* against a human breast cancer cell line (MCF-7) was evaluated in vitro using MTT assays, nuclear morphological characteristics, and RT-PCR and western blot analyses. MCF-7 cells treated with either AgNPs or cisplatin exhibited decreased Bcl-2 expression and increased Bax expression, indicating the involvement of mitochondria in the mechanism of death induced by AgNPs [101].

Mitochondria function as critical centers of signaling; their integrity can be compromised by various regulators of apoptosis [102, 103]. The generation of ROS by AgNPs may also require mitochondria, which may initiate intrinsic caspase-dependent apoptotic pathways leading to cell death. Nanoparticles synthesized with *Rosa indica* extracts have the potential to be used in a wide range of therapeutic anticancer applications. AgNPs synthesized with green petals of *R. indica* act as radical scavengers and induce apoptosis in HCT-15 cells and the generation of ROS [104].

#### Antioxidant Activity of AgNPs

AgNPs synthesized using *Leptadenia reticulata* leaf extracts, at a concentration of 500 μg/mL, have the highest recorded radical scavenging activity of 64.81 % [105]. Plant extracts promote DPPH radical scavenging activity of AgNPs, which is dose dependent. The ability of antioxidants to scavenge DPPH radicals is likely due to their ability to donate hydrogens and easily incorporate electrons; the latter is possible due to the presence of host lipophilic radicals. A change of color from purple to yellow was observed at 517 nm [106]. The DPPH radical scavenging activity of HAuCl_4_ and AgNO_3_ was trivial compared to nanoparticles, which may be due to salt conditions or weaker solubility of metal oxides [107].

A sophisticated reaction can be observed between phenolic compounds and phosphotungstic and phosphomolybdic acids in Folin-Ciocalteu reagents [108]. Phenolic compounds present in plant extracts exhibited high antioxidant and reduction activities, which are important for the synthesis of AgNPs [109]. The higher total phenolic content of *Eclipta prostrata* leaf extracts supports the assembly of silver ions into smaller AgNPs, because of the donation of electrons by these compounds [110].

#### Antidiabetic Activity of AgNPs

The ability of AgNPs synthesized using stem extracts of *Tephrosia tinctoria* to control blood sugar levels was evaluated. AgNPs scavenged free radicals, decreased levels of enzymes that catalyze the hydrolysis of complex carbohydrates (α-glucosidase and α-amylase), and increased the consumption rate of glucose [111]. Due to the adverse effects of methylene blue (MB) on the environment, the removal of MB from wastewater is an important area of research and a key challenge for researchers. AgNPs synthesized with aqueous stem extracts of *Salvadora persica* were able to degrade MB in a light-dependent manner; by converting hazardous materials into nonhazardous ones, AgNPs potentially have significant applications in water purification [112]. MB exhibits characteristic absorption peaks at 663 and 614 nm, which were used to monitor the photo-degradation of MB. Higher concentrations induce the aggregation of AgNPs, leading to an increase in particle size and a decrease in specific surface area and surface active sites of particles [113]. The most effective concentration of AgNPs for the photo-degradation of MB was 8 mg.

#### Different Field Applications of AgNPs

**Fig. 4:**
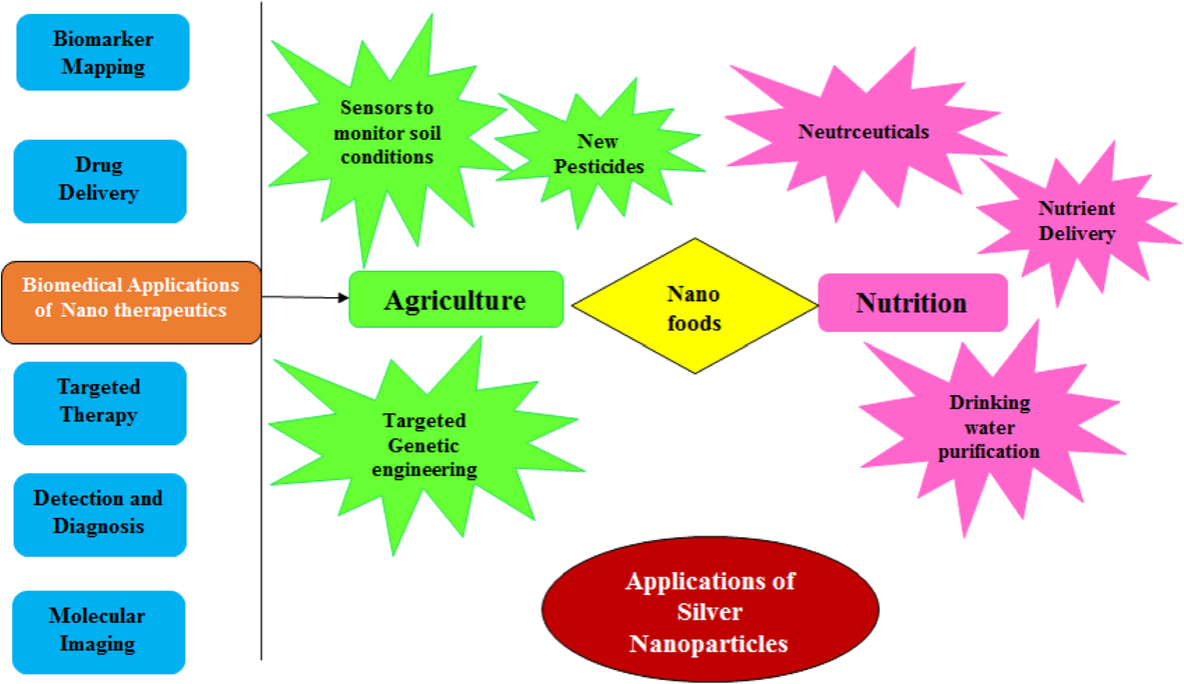
Different applications of synthesized silver nanoparticles

Nanotechnology applications are highly suited for biological molecules because of their unique properties. Nanotechnology is a growing area of research in the fields of material science and biological science [114]. Silver nanoparticles have attracted the attention of researchers because of their broad applications in diverse areas, such as integrated circuits [115], sensors [116], biolabeling, filters, antimicrobial deodorant fibers [117], cell electrodes [43], low-cost paper batteries (silver nano-wires) [118], and antimicrobials [119]. AgNPs have been used extensively as antimicrobial agents in the health industry, food storage, textile coatings, and a number of environmental applications [120], few of which are shown in Fig. 4. Antimicrobial properties of AgNPs are beneficial for different fields of medicine, various industries, animal husbandry, packaging, accessories, cosmetics, health, and the military. In general, therapeutic effects of silver particles (in suspension) depend on different parameters, including particle size (surface area and energy), particle shape (catalytic activity), particle concentration (therapeutic index), and particle charge (oligodynamic quality) [121].

The viability of A549 cells treated for 6 h with 10 and 50 μg/mL of AgNPs synthesized with *Albizia adianthifolia* leaf extracts was 21 and 73 %, respectively, and that of normal peripheral lymphocytes was 117 and 109 %, respectively, indicating that AgNPs are nontoxic to normal PLs cells [122]. AgNPs synthesized with *Indigofera aspalathoides* extracts were tested in wound-healing applications following excision in animal models [28]. AgNPs synthesized with *Chrysanthemum morifolium* extracts were added to clinical ultrasound gels, which are used with an ultrasound probe, and were found to have bactericidal activity, contributing to the sterility of the instrument [123]. AgNPs synthesized with *M. zapota* leaf extracts exhibited acaricidal activity against *Rhipicephalus* (*Boophilus*) *microplus* (LC_50_ = 3.44 mg/L) [58]. The IC_50_ values of AgNPs synthesized using aqueous extracts of Ashoka or neem leaves against *Plasmodium falciparum* were 8 and 30 μg/mL, respectively [124]. Appreciable larvicidal activity of AgNPs synthesized with aqueous extracts of *E. prostrata* was observed against *Anopheles subpictus* and *Culex tritaeniorhynchus* [125]. The population of bacteria decreased after 6 h when 10 mg of AgNPs synthesized using *P. juliflora* leaf extracts was used to treat 100 mL of sewage and increased over time [56]. AgNPs synthesized with *Acacia nilotica* pod extracts were used to treat glassy carbon electrodes, which exhibited greater catalytic activity in reducing benzyl chloride than glassy carbon and metallic Ag electrodes [126]. AgNPs synthesized using *Gloriosa superba* extracts act through the electron relay effect and influence the degradation of MB after 30 min [127].

## Conclusions

In summary, silver nanoparticles (AgNPs) exhibit remarkable physical, mixture, optical, and natural properties compared to other biomedical nanomaterials, which make them ideal in various stages of diverse biomedical applications. Nanoparticles synthesized with plant concentrates have yielded promising results in biomedical applications. Comprehensive examination further contemplated that repercussions of nanoparticles give essentially the same number of preferences and purposes of enthusiasm for remedial applications in examination to standard medicines and antidotes to poisons. The use of AgNPs in medicinal transport systems may also be utilized in the future in the field of arrangement. AgNPs have the potential to function as therapeutics with diverse clinical and pharmacological properties. They may be used in broad applications, including as anticancer agents or bactericidal agents during surgery or recovery. In this way, the green synthesis of AgNPs as novel remedial authorities will be significant in various biomedical applications. Notwithstanding their potential in restorative applications, the impact of AgNPs on human welfare (both positive and negative) should be completely considered before their widespread use. The adaptability of manufacturing techniques for AgNPs and their easy reconstitution into distinct media have prompted further research into the hypothetical impact of nanoparticles as antimicrobial, antiviral, and mitigating agents. The shape, size, and size distribution of AgNPs can be controlled by modifying synthesis conditions, such as with specialists, stabilizers, or distinct engineering techniques. The productive translation of silver into nanotechnology applications requires safe, creative, and eco-conscious strategies and greater control over their biodistribution and pharmacokinetics in clinical applications.

## References

[1] Albrecht MA, Evans CW, Raston CL (2006). Green chemistry and the health implications of nanoparticles. Green Chem.

[2] Rai M, Yadav A, Gade A (2008). Current trends in phytosynthesis of metal nanoparticles. Crit Rev Biotechnol.

[3] Sinha S, Pan I, Chanda P, Sen SK (2009). Nanoparticles fabrication using ambient biological resources. J Appl Biosci.

[4] Bhattacharyya A, Bhaumik A, Rani PU, Mandal S, Epidi TT (2010). Nanoparticles—a recent approach to insect pest control. Afr J Biotechnol.

[5] Huang J, Chen C, He N, Hong J, Lu Y, Qingbiao L (2007). Biosynthesis of silver and gold nanoparticles by novel sundried *Cinnamomum camphora* leaf. Nanotechnology.

[6] Dipankar C, Murugan S (2012). The green synthesis, characterization and evaluation of the biological activities of silver nanoparticles synthesized from *Iresine herbstii* leaf aqueous extracts. Colloids Surf B: Biointerfaces.

[7] Becker RO, Spadaro JA (1978). Treatment of orthopaedic infections with electrically generated silver ions. J Bone Joint Surg.

[8] Liu Z, Bucknall DG, Allen MG (2011). Inclined nanoimprinting lithography for 3D nanopatterning. Nanotechnology.

[9] Mohanpuria P, Rana NK, Yadav SK (2008). Biosynthesis of nanoparticles: technological concepts and future applications. J Nanoparticle Res.

[10] Guzmán MG, Dille J, Godet S (2009). Synthesis of silver nanoparticles by chemical reduction method and their antibacterial activity. Int J Chem Biomol Eng.

[11] Rodríguez-Sánchez ML, Blanco MC, López-Quintela MA (2000). Electrochemical synthesis of silver nanoparticles. J Phys Chem B.

[12] Sharma VK, Yngard RA, Lin Y (2009). Silver nanoparticles green synthesis and their antimicrobial activities. Adv Colloid Interf Sci.

[13] Goodsell DS (2004). Bionanotechnology: lessons from nature.

[14] Abdel-Halim ES, El-Rafie MH, Al-Deyab SS (2011). Polyacrylamide/guar gum graft copolymer for preparation of silver nanoparticles. Carbohydr Polym.

[15] Yokohama K, Welchons DR (2007). The conjugation of amyloid beta protein on the gold colloidal nanoparticles surfaces. Nanotechnology.

[16] Song JY, Kim BS (2009). Rapid biological synthesis of silver nanoparticles using plant leaf extracts. Bioprocess Biosyst Eng.

[17] Mohapatra B, Kuriakose S, Mohapatra S (2015). Rapid green synthesis of silver nanoparticles and nanorods using *Piper nigrum* extract. J Alloys and Compounds.

[18] Vigneshwaran N, Kathe AA, Varadarajan PV, Nachane RP, Balasubramanya RJ (2007). Functional finishing of cotton fabrics using silver nanoparticles. J Nanosci Nanotechnol.

[19] Chaudhry Q, Castle L (2011). Food applications of nanotechnologies: an overview of opportunities and challenges for developing countries. Trends Food Sci Technol.

[20] Martinez-Gutierrez F, Olive PL, Banuelos A, Orrantia E, Nino N, Sanchez E (2010). Synthesis, characterization, and evaluation of antimicrobial and cytotoxic effect of silver and titanium nanoparticles. Nanomedicine.

[21] Kokura S, Handa O, Takagi T, Ishikawa T, Naito Y, Yoshikawa T (2010). Silver nanoparticles as a safe preservative for use in cosmetics. Nanomedicine.

[22] Shankar SS, Rai A, Ankamwar B, Singh A, Ahmad A, Sastry M (2004). Biological synthesis of triangular gold nanoprisms. Nat Mater.

[23] Shankar SS, Ahmad A, Pasricha R, Sastry M (2003). Bioreduction of chloroaurate ions by geranium leaves and its endophytic fungus yields gold nanoparticles of different shapes. J Mater Chem.

[24] Shankar SS, Ahmad A, Sastry M (2003). Geranium leaf assisted biosynthesis of silver nanoparticles. Biotechnol Prog.

[25] Rai A, Singh A, Ahmad A, Sastry M (2006). Role of halide ions and temperature on the morphology of biologically synthesized gold nanotriangles. Langmuir.

[26] Rai A, Chaudhary M, Ahmad A, Bhargava S, Sastry M (2007). Synthesis of triangular Au core-Ag shell nanoparticles. Mater Res Bull.

[27] Chandran SP, Chaudhary M, Pasricha R, Ahmad A, Sastry M (2006). Synthesis of gold nanotriangles and silver nanoparticles using *Aloe vera* plant extract. Biotechnol Prog.

[28] Arunachalam KD, Annamalai SK, Hari S (2013). One-step green synthesis and characterization of leaf extract-mediated biocompatible silver and gold nanoparticles from *Memecylon umbellatum*. Int J Nanomed.

[29] Mittal AK, Tripathy D, Choudhary A, Aili PK, Chatterjee A, Singh IP, Banerjee UC (2015). Bio-synthesis of silver nanoparticles using *Potentilla fulgens* Wall. ex Hook. and its therapeutic evaluation as anticancer and antimicrobial agent. Mater Sci Eng C Mater Biol Appl.

[30] Ledwith DM, Whelan AM, Kelly JM (2007). A rapid, straight-forward method for controlling the morphology of stable silver nanoparticles. J Mater Chem.

[31] Tripathi GNR (2003). p-Benzosemiquinone radical anion on silver nanoparticles in water. J Am Chem Soc.

[32] Zhang J, Malicka J, Gryczynski I, Lakowicz JR (2005). Surface-enhanced fluorescence of fluorescein-labeled oligonucleotides capped on silver nanoparticles. J Phys Chem B.

[33] Aroca RF, Alvarez-Puebla RA, Pieczonka N, Sanchez-Cortez S, Garcia-Ramos JV (2005). Surface-enhanced Raman scattering on colloidal nanostructures. Adv Colloid Interf Sci.

[34] Jiang ZJ, Liu CY, Sun LW (2005). Catalytic properties of silver nanoparticles supported on silica spheres. J Phys Chem B.

[35] Atiyeh BS, Costagliola M, Hayek SN, Dibo SA (2007). Effect of silver on burn wound infection control and healing: review of the literature. Burns.

[36] Lokini S, Narayanan V (2013). Antimicrobial and anticancer activity of gold nanoparticles synthesized from grapes fruit extract. Chem SciTrans.

[37] Gericke M, Pinches A (2006). Microbial production of gold nanoparticles. Gold Bull.

[38] Bai HJ, Zhang ZM, Guo Y, Yang GE (2009). Biosynthesis of cadmium sulfide nanoparticles by photosynthetic bacteria *Rhodopseudomonas palustris*. Colloids Surf B Biointerf.

[39] Aromal SA, Philip D (2012). Green synthesis of gold nanoparticles using *Trigonella foenum*-*graecum* and its size dependent catalytic activity. Spectrochim Acta A.

[40] Dubey SP, Lahtinen M, Särkkä H, Sillanpää M (2010). Bioprospective of Sorbus aucuparia leaf extract in development of silver and gold nanocolloids. Colloid Surf B.

[41] Li S, Shen Y, Xie A, Yu X, Qiu L, Zhang L, Zhang Q (2007). Green synthesis of silver nanoparticles using *Capsicum annuum* L. extract. Green Chem.

[42] Shameli K, Ahmad M, Al-Mulla EAJ, Ibrahim NA (2012). Green biosynthesis of silver nanoparticles using *Callicarpa maingayi* stem bark extraction. Molecules.

[43] Bhattacharya D, Gupta RK (2005). Nanotechnology and potential of microorganisms. Crit Rev Biotechnol.

[44] Ankanna S, Prasada TNVKV, Elumalai EKB, Savithramma N (2010). Production of biogenic silver nanoparticles using *Boswellia ovalifoliolata* stem bark. Dig J Nanomater Biostruct.

[45] Gardea-Torresdey JL, Tiemann KJ, Gamez G, Dokken K, Tehuacanero S, Jose-Yacaman M (1999). Gold nanoparticles obtained by bio-precipitation from gold(III) solutions. J Nanoparticle Res.

[46] Mock JJ, Barbic M, Smith DR, Schultz DA, Schultz SJ (2002). Shape effect in palms resonance of individual colloidal silver nanoparticles. J Chem Phys.

[47] Korbekandi H, Iravani S, Abbasi S (2009). Production of nanoparticles using organism’s production of nanoparticles using organisms. Critical Rev Biotechnol.

[48] Klaus-Joerger T, Joerger R, Olsson E, Granqvist C (2001). Bacteria as workers in the living factory: metal-accumulating bacteria and their potential for materials science. Trends Biotechnol.

[49] Dwivedi AD, Gopal K (2010). Biosynthesis of gold and silver nanoparticles using *Chenopodium album* leaf extract. Coll Surf A.

[50] Veerasamy R, Xin TZ, Gunasagaran S, Xiang TFW, Yang EFC, Jeyakumar N (2011). Biosynthesis of silver nanoparticles using mangosteen leaf extract and evaluation of their antimicrobial activities. J Saudi Chem Soci.

[51] Iravani S, Zolfaghari B (2013). Green synthesis of silver nanoparticles using *Pinus eldarica* bark extract. BioMed Res Int.

[52] Sulaiman GM, Mohammed WH, Marzoog TR, Al-Amiery AA, Kadhum AA, Mohamad AB, Bagnati R (2013). Green synthesis, antimicrobial and cytotoxic effects of silver nanoparticles using *Eucalyptus chapmaniana* leaves extract. Asian Pac J Trop Biomed.

[53] Thirunavoukkarasu M, Balaji U, Behera S, Panda PK, Mishra BK (2013). Biosynthesis of silver nanoparticle from leaf extract of *Desmodium gangeticum* (L.) DC. and its biomedical potential. Spectrochim Acta A Mol Biomol Spectrosc.

[54] Vilchis-Nestor AR, Sánchez-Mendieta V, Camacho-López MA, Gómez-Espinoza RM, Camacho-López MA, Arenas-Alatorre J (2008). Solventless synthesis and optical properties of Au and Ag nanoparticles using *Camellia sinensis* extract. Mater Lett.

[55] Singhal G, Bhavesh R, Kasariya K, Sharma AR, Singh RP (2011). Biosynthesis of silver nanoparticles using *Ocimum sanctum* (Tulsi) leaf extract and screening its antimicrobial activity. J Nanopart Res.

[56] Kora AJ, Sashidhar RB, Arunachalam J (2010). Gum kondagogu (*Cochlospermum gossypium*): a template for the green synthesis and stabilization of silver nanoparticles with antibacterial application. Carbohydr Polym.

[57] Reddy NJ, Vali DN, Rani M, Rani SS (2014). Evaluation of antioxidant, antibacterial and cytotoxic effects of green synthesized silver nanoparticles by *Piper longum* fruit. Mat Sci Eng C.

[58] Rajakumar G, Rahuman AA (2012). Acaricidal activity of aqueous extract and synthesized silver nanoparticles from *Manilkara zapota* against *Rhipicephalus* (*Boophilus*) *microplus*. Res Vet Sci.

[59] Morais PC, Santos RL, Pimenta ACM, Azevedo RB, Lima ECD (2006). Preparation and characterization of ultra-stable biocompatible magnetic fluids using citrate coated cobalt ferrite nanoparticles. Thin Sol Fil.

[60] Alexandrova K, Markova-Deneva I, Gigova A, Dragieva I (2008) In: Dimov S, Menz W (eds) TEM/SEM and FT-IR characterization of biocompatible magnetic nanoparticles. Multi-Material Micro Manufacture., pp 1–4, Cardiff University, Cardiff, UK: Published by Whittles Publishing Ltd.

[61] Raja K, Saravanakumar A, Vijayakumar R (2012). Efficient synthesis of silver nanoparticles from *Prosopis juliflora* leaf extract and its antimicrobial activity using sewage. Spectrochim Acta A Mol Biomol Spectrosc.

[62] Suriyakalaa U, Antony JJ, Suganya S, Siva D, Sukirtha R, Kamalakkannan S, Pichiah T, Achiraman S (2013). Hepatocurative activity of biosynthesized silver nanoparticles fabricated using *Andrographis paniculata*. Coll Surf B.

[63] Singh C, Baboota RK, Naik PK, Singh H (2012). Biocompatible synthesis of silver and gold nanoparticles using leaf extract of *Dalbergia sissoo*. Adv Mater Lett.

[64] Ajitha B, Reddy YAK, Reddy PS (2015). Green synthesis and characterization of silver nanoparticles using *Lantana camara* leaf extract. Mater Sci Eng C.

[65] Gavade NL, Kadam AN, Suwarnkar MB, Ghodake VP, Garadkar KM (2015). Biogenic synthesis of multi-applicative silver nanoparticles by using *Ziziphus Jujuba* leaf extract. Spectrochim Acta A Mol Biomol Spectrosc.

[66] Khandelwal N, Singh A, Jain D, Upadhyay MK, Verma HN (2010). Green synthesis of silver nanoparticles using *Argemone mexicana* leaf extract and evaluation of their activity. Digest J Nanomater Biostruct.

[67] Jha AK, Prasad K (2010). Green synthesis of silver nanoparticles using *Cycas* leaf. Int J Green Nanotech Phy Chem.

[68] Edison TJI, Sethuraman MG (2012). Instant green synthesis of silver nanoparticles using *Terminalia chebula* fruit extract and evaluation of their catalytic activity on reduction of methylene blue. Pro Biochem.

[69] Zargar M, Shameli K, Najafi GR, Farahani F (2014). Plant mediated green biosynthesis of silver nanoparticles using Vitex negundo L extract. J Ind & Eng Chem.

[70] Khan M, Khan M, Adil SF, Tahir MN, Tremel W, Alkhathlan HZ, Al-Warthan A, Siddiqui MR (2013). Green synthesis of silver nanoparticles mediated by *Pulicaria glutinosa* extract. Int J Nanomed.

[71] Jagtap UB, Bapat VA (2013). Green synthesis of silver nanoparticles using *Artocarpus heterophyllus* Lam. seed extract and its antibacterial activity. Ind Crops Prod.

[72] Kumar PPNV, Kollu SVNPP, Satyanarayan KVV, Shameem U (2014). Green synthesis and characterization of silver nanoparticles using *Boerhaavia diffusa* plant extract and their antibacterial activity. Ind Crops Prod.

[73] Kapil A (2005). The challenge of antibiotic resistance: need to contemplate. Ind J Med Res.

[74] Marambio-Jones C, Hoek EMV (2010). A review of the antibacterial effects of silver nanomaterials and potential implications for human health and the environment. J Nanopart Res.

[75] Monteiro DR, Gorup LF, Takamiya AS, Ruvollo-Filho AC, de Camargo ER, Barbosa DB (2009). The growing importance of materials that prevent microbial adhesion: antimicrobial effect of medical devices containing silver. Int J Antimicrob Agents.

[76] Kvitek DJ, Will JL, Gasch AP (2008). Variation in stress sensitivity and genomic expression in diverse *S. cerevisiae* isolates. PLoS Genet.

[77] Kim JS, Kuk E, Yu KN, Kim JH, Park SJ (2007). Antimicrobial effects of silver nanoparticles. Nanomed Nanotechnol Biol Med.

[78] Dibrov P, Dzioba J, Gosink KK, Hase CC (2002). Chemiosmotic mechanism of antimicrobial activity of Ag (+) in *Vibrio cholerae*. Antimicrob Agents Chemother.

[79] Song HY, Ko KK, Oh IH, Lee BT (2006). Fabrication of silver nanoparticles and their antimicrobial mechanisms. Europ Cells Mat.

[80] Feng QL, Wu J, Chen GQ, Cui FZ, Kim TN, Kim JO (2000). A mechanistic study of the antibacterial effect of silver ions on Escherichia coli and *Staphylococcus aureus*. J Biomed Mat Res.

[81] Ahmed KBA, Subramanian S, Sivasubramanian A, Veerappan G, Veerappan A (2014). Preparation of gold nanoparticles using *Salicornia brachiata* plant extract and evaluation of catalytic and antibacterial activity. Spectrochimica Acta Part A Molecul Biomol Spectros.

[82] Gnanadesigan M, Anand M, Ravikumar S, Maruthupandy M (2011). Biosynthesis of silver nanoparticles by using mangrove plant extract and their potential mosquito property. Asian Pac J Trop Med.

[83] Sankar R, Karthik A, Prabu A, Karthik S, Shivashangari KS, Ravikumar V (2013). *Origanum vulgare* mediated biosynthesis of silver nanoparticles for its antibacterial and anticancer activity. Coll Surf B Biointer.

[84] Sathishkumar G, Gobinath C, Karpagam K, Hemamalini V, Premkumar K, Sivaramakrishnan S (2012). Phyto-synthesis of silver nanoscale particles using *Morinda citrifolia* L. and its inhibitory activity against human pathogens. Colloids Surf B: Biointerfaces.

[85] Ghaedi M, Yousefinejad M, Safarpoor M, Zare Khafri H, Purkait MK (2015). *Rosmarinus officinalis* leaf extract mediated green synthesis of silver nanoparticles and investigation of its antimicrobial properties. J Ind Eng Chem.

[86] Nezamdoost T, Bagherieh-Najjar MB, Aghdasi M (2014). Biogenic synthesis of stable bioactive silver chloride nanoparticles using *Onosma dichroantha* Boiss. Root extract. Mater Lett.

[87] Velmurugan P, Choa M, Lim SS, Seo SK (2015). Phytosynthesis of silver nanoparticles by *Prunus yedoensis* leaf extract and their antimicrobial activity. Mater Lett.

[88] Saxena A, Tripathi RM, Zafar F, Singh P (2012). Green synthesis of silver nanoparticles using aqueous solution of *Ficus benghalensis* leaf extract and characterization of their antibacterial activity. Mater Lett.

[89] Tripathi RM, Saxena A, Gupta N, Kapoor H, Singh RP (2010). High antibacterial activity of silver nanoballs against *E. Coli* MTCC 1302, *S. typhimurium* MTCC 1254, *B. subtilis* MTCC 1133 and *P. aeruginosa* MTCC 2295. Digest J Nanomater Biostruct.

[90] Hajipour MJ, Fromm KM, Ashkarran AA, Aberasturi DJD (2012). Antibacterial properties of nanoparticles. Trends Biotechnol.

[91] MubarakAli D, Thajuddin N, Jeganathan K, Gunasekaran M (2011). Plant extract mediated synthesis of silver and gold nanoparticles and its antibacterial activity against clinically isolated pathogens. Colloids Surf B.

[92] Azam A, Ahmed F, Arshi N, Chaman M, Naqvi AH (2009). One step synthesis and characterization of gold nanoparticles and their antibacterial activities against *E. coli* (ATCC 25922 strain). Int J Theor Appl Sci.

[93] AshaRani PV, Mun GLK, Hande MP, Valiyaveettil S (2009). Cytotoxicity and genotoxicity of silver nanoparticles in human cells. ACS Nano.

[94] Morones JR, Elechiguerra LJ, Camacho A, Holt K, Kouri BJ, Ramirez TJ (2005). The bactericidal effect of silver nanoparticles. Nanotechnology.

[95] Das J, Paul Das M, Velusamy P (2013). *Sesbania grandiflora* leaf extract mediated green synthesis of antibacterial silver nanoparticles against selected human pathogens. Spectrochim Acta A Mol Biomol Spectrosc.

[96] Balakumaran MD, Ramachandran R, Kalaichelvan PT (2015). Exploitation of endophytic fungus, *Guignardia mangiferae* for extracellular synthesis of silver nanoparticles and their in vitro biological activities. Microbiol Res.

[97] Nayak D, Pradhan S, Ashe S, Rauta PR, Nayak B (2015). Biologically synthesized silver nanoparticles from three diverse family of plant extracts and their anticancer activity against epidermoid A431 carcinoma. J Colloid Interface Sci.

[98] Das S, Das J, Samadder A, Bhattacharyya SS, Das D, Khuda-Bukhsh AR (2013). Biosynthesized silver nanoparticles by ethanolic extracts of *Phytolacca decandra*, *Gelsemium sempervirens*, *Hydrastis canadensis* and *Thuja occidentalis* induce differential cytotoxicity through G2/M arrest in A375 cells. Colloids Surf B: Biointerfaces.

[99] Subramanian V, Suja S (2012). Green synthesis of silver nanoparticles using Coleus amboinicus lour, antioxidant activity and in vitro cytotoxicity against Ehrlich’s ascite carcinoma. Indian J Med Res Pharm Sci.

[100] Gogoi N, Babu PJ, Mahanta C, Bora U (2015). Green synthesis and characterization of silver nanoparticles using alcoholic flower extract of *Nyctanthes arbor-tristis* and *in vitro* investigation of their antibacterial and cytotoxic activities. Mater Sci Eng C Mater Biol Appl.

[101] Ramar M, Manikandan B, Marimuthu PN, Raman T (2015). Synthesis of silver nanoparticles using *Solanum trilobatum* fruits extract and its antibacterial, cytotoxic activity against human breast cancer cell line MCF 7. Spectrochim Acta A Mol Biomol Spectrosc.

[102] Green DR, Reed JC (1998). Mitochondria and apoptosis. Science.

[103] Kroemer G, Zamzami N, Susin SA (1997). Mitochondrial control of apoptosis. Immunol Today.

[104] Manikandan R, Manikandan B, Raman T, Arunagirinathan K (2015). Biosynthesis of silver nanoparticles using ethanolic petals extract of *Rosa indica* and characterization of its antibacterial, anticancer and anti-inflammatory activities. Spectrochim Acta A Mol Biomol Spectrosc.

[105] Swamy MK, Sudipt KM, Jayant K, Balasubramany S (2014). The green synthesis, characterization, and evaluation of the biological activities of silver nanoparticles synthesized from *Leptadenia reticulata* leaf extract. App Nanosci.

[106] Baumann J, Wurn G, Bruchlausen FV (1979). Prostaglandin synthetase inhibiting O_2_ radical scavenging properties of some flavonoids and related phenolic compounds. Arch Pharmacol.

[107] Nie Z, Liu KJ, Zhong CJ, Wang LF, Yang Y, Tian Q, Liu Y (2007). Enhanced radical scavenging activity by antioxidant-functionalized gold nanoparticles: a novel inspiration for development of new artificial antioxidants. Free Radic Biol Med.

[108] Nguyen PM, Kwee EM, Niemeyer ED (2010). Potassium rate alters the antioxidant capacity and phenolic concentration of basil *Ocimum basilicum* L. leaves. Food Chem.

[109] Quaresma P, Soares L, Contar L, Miranda A, Osorio I, Carvalho PA, Franco R, Pereira E (2009). Green photocatalytic synthesis of stable Au and Ag nanoparticles. Green Chem Comm.

[110] Wang W, Chen Q, Jiang C, Yang D, Liu X, Xu S (2007). One step synthesis of biocompatible gold nanoparticles using gallic acid in the presence of poly-(N-vinyl-2-pyrrolidone). Colloids Surf A Physicochem Eng Asp.

[111] Rajaram K, Aiswarya DC, Sureshkumar P (2015). Green synthesis of silver nanoparticle using *Tephrosia tinctoria* and its antidiabetic activity. Mater Lett.

[112] Tahir K, Nazir S, Li B, Khan AU (2015). An efficient photo catalytic activity of green synthesized silver nanoparticles using *Salvadora persica* stem extract. Sep Purif Technol.

[113] Zhao D, Wang J, Zhang Z, Zhang J (2009). Photocatalytic degradation of omethoate using NaY zeolite-supported TiO_2_. Front Chem Eng China.

[114] Kaviya S, Santhanalakshmi J, Viswanathan B, Muthumary J, Srinivasan K (2011). Biosynthesis of silver nanoparticles using *citrus sinensis* peel extract and its antibacterial activity. Spectrochim Acta A Mol Biomol Spectrosc.

[115] Kasthuri J, Veerapandian S, Rajendiran N (2009). Biological and synthesis of silver and gold nanoparticles using apiin as reducing agent. Colloids Surf B: Biointerfaces.

[116] Kotthaus SG, Hang BH, Schafer H (1997). Study of isotropically conductive bondings filled with aggregates of nano-sited Ag-particles. IEEE Trans Compon Packaging Technol.

[117] Zhang WWG (2003). Research and development for antibacterial materials of silver nanoparticle. New Chem Mater.

[118] Hong KHP, Park JL, Sul IH, Youk JH, Kang TJ (2006). Preparation of antimicrobial poly(vinyl alcohol) nanofibers containing silver nanoparticles. J Polym Sci Part B Polym Phys.

[119] Cho KH, Park JE, Osaka T, Park SG (2005). The study of antimicrobial activity and preservative effects of nanosilver ingredient. Electrochim Acta.

[120] Duran N, Marcato DP, De Souza HI, Alves LO, Espsito E (2007). Antibacterial effect of silver nanoparticles produced by fungal process on textile fabrics and their effluent treatment. J Biomedical Nanotechnology.

[121] Yoon KY, Hoon Byeon J, Park JH, Hwang J (2007). Susceptibility constants of *Escherichia coli* and *Bacillus subtilis* to silver and copper nanoparticles. Sci Total Environ.

[122] Gengan RM, Anand K, Phulukdaree A, Chuturgoon A (2013). A549 lung cell line activity of biosynthesized silver nanoparticles using *Albizia adianthifolia* leaf. Colloids Surf B Biointerf.

[123] He Y, Du Z, Lv H (2013). Green synthesis of silver nanoparticles by *Chrysanthemum morifolium* Ramat. Extract and their application in clinical ultrasound gel. Int J Nanomed.

[124] Mishra A, Kaushik NK, Sardar M, Sahal D (2013). Evaluation of antiplasmodial activity of green synthesized silver nanoparticles. Colloids Surf B Biointerf.

[125] Rajakumar G, Rahuman AA (2011). Larvicidal activity of synthesized silver nanoparticles using *Eclipta prostrata* leaf extract against filariasis and malaria vectors. Acta Trop.

[126] Jebakumar TN, Edison I, Sethuraman MG (2013). Electrocatalytic reduction of benzyl chloride by green synthesized silver nanoparticles using pod extract of *Acacia nilotica*. ACS Sustain Chem Eng.

[127] Ashokkumar S, Ravi S, Velmurugan S (2013). Green synthesis of silver nanoparticles from *Gloriosa superba* L. leaf extract and their catalytic activity. Spectrochim Acta A Mol Biomol Spectrosc.

[128] Sana SS, Badineni VR, Arla SK, Boya VKN (2015). Eco-friendly synthesis of silver nanoparticles using leaf extract of *Grewia flaviscences* and study of their antimicrobial activity. Mater Lett.

[129] Raman RP, Parthiban S, Srinithya B, Vinod Kumar V, Anthony SP, Sivasubramanian A, Muthuraman MS (2015). Biogenic silver nanoparticles synthesis using the extract of the medicinal plant *Clerodendrum serratum* and its *in*-*vitro* antiproliferative activity. Mater Lett.

[130] Mishra PM, Sahoo SK, Naik GK, Parida N (2015). Biomimetic synthesis, characterization and mechanism of formation of stable silver nanoparticles using *Averrhoa carambola* L. leaf extract. Mater Lett.

[131] Banala RR, Nagati VB, Karnati PR (2015). Green synthesis and characterization of *Carica papaya* leaf extract coated silver nanoparticles through X-ray diffraction, electron microscopy and evaluation of bactericidal properties. Saudi J Bio Sci.

[132] Kumar B, Smita K, Cumbal L, Debut A (2014). Synthesis of silver nanoparticles using Sacha inchi (*Plukenetia volubilis* L.) leaf extracts. Saudi J Bio Sci.

[133] Murugan K, Labeeba MA, Panneerselvam C, Dinesh D (2015). *Aristolochia indica* green-synthesized silver nanoparticles: a sustainable control tool against the malaria vector *Anopheles stephensi*. Res Vet Sci.

[134] Nasrollahzadeh M, Sajadi SM, Babaei F, Mahamd M (2015). *Euphorbia helioscopia* Linn as a green source for synthesis of silver nanoparticles and their optical and catalytic properties. J Colloid Interface Sci.

[135] Murugan K, Dinesh D, Kumar PJ (2015). Datura metal-synthesized silver nanoparticles magnify predation of dragonfly nymphs against the malaria vector *Anopheles stephensi*. Parasitol Res.

[136] Swamy MK, Akhtar MS, Mohanty SK, Sinniah UR (2015). Synthesis and characterization of silver nanoparticles using fruit extract of *Momordica cymbalaria* and assessment of their *in vitro* antimicrobial, antioxidant and cytotoxicity activities. Spectrochim Acta A Mol Biomol Spectrosc.

[137] Roni M, Murugan K, Panneerselvam C, Subramaniam J, Nicoletti M (2015). Characterization and biotoxicity of *Hypnea musciformis*-synthesized silver nanoparticles as potential eco-friendly control tool against *Aedes aegypti* and *Plutella xylostella*. Ecotoxicol Environ Saf.

[138] Santhosh SB, Yuvarajan R, Natarajan D (2015). *Annona muricata* leaf extract-mediated silver nanoparticles synthesis and its larvicidal potential against dengue, malaria and filariasis vector. Parasitol Res.

[139] Bose D, Chatterjee S (2015). Antibacterial activity of green synthesized silver nanoparticles using Vasaka (*Justicia adhatoda* L.) leaf extract. Indian J Microbiol.

[140] Latha M, Sumathi M, Manikandan R, Arumugam A, Prabhu NM (2015). Biocatalytic and antibacterial visualization of green synthesized silver nanoparticles using *Hemidesmus indicus*. Microb Pathog.

[141] Ramesh PS, Kokila T, Geetha D (2015). Plant mediated green synthesis and antibacterial activity of silver nanoparticles using *Emblica officinalis* fruit extract. Spectrochim Acta A Mol Biomol Spectrosc.

[142] Korbekandi H, Chitsazi MR, Asghari G, Bahri Najafi R, Badii A, Iravani S (2015). Green biosynthesis of silver nanoparticles using *Quercus brantii* (oak) leaves hydroalcoholic extract. Pharm Bio.

[143] Bhakya S, Muthukrishnan S, Sukumaran M, Muthukumar M (2015). Biogenic synthesis of silver nanoparticles and their antioxidant and antibacterial activity. Appl Nanosci.

[144] Perugu S, Nagati V, Bhanoori M (2015). Green synthesis of silver nanoparticles using leaf extract of medicinally potent plant Saraca indica: a novel study. Appl Nanosci.

[145] Prathap M, Alagesan A, Ranjitha Kumari BD (2014). Anti-bacterial activities of silver nanoparticles synthesized from plant leaf extract of *Abutilon indicum* (L.) wweet. J Nanostruct Chem.

[146] Miri A, Sarani M, Rezazade Bazaz M, Darroudi M (2015). Plant-mediated biosynthesis of silver nanoparticles using *Prosopis farcta* extract and its antibacterial properties. Spectrochim Acta A Mol Biomol Spectrosc.

[147] Chitra G, Balasubramani G, Ramkumar R, Sowmiya R, Perumal P (2015). *Mukia maderaspatana* (Cucurbitaceae) extract-mediated synthesis of silver nanoparticles to control *Culex quinquefasciatus* and *Aedes aegypti* (Diptera: Culicidae). Parasitol Res.

[148] Borase HP, Salunkhe RB, Patil CD, Suryawanshi RK, Salunke BK, Wagh ND, Patil SV (2015). Innovative approach for urease inhibition by *Ficus carica* extract-fabricated silver nanoparticles: an *in vitro* study. Biotechnol Appl Biochem.

[149] Khatami M, Pourseyedi S, Khatami M, Hamidi H, Zaeifi M, Soltani L (2015). Synthesis of silver nanoparticles using seed exudates of *Sinapis arvensis* as a novel bioresource, and evaluation of their antifungal activity. Bioresources and Bioprocessing.

[150] Rajkuberan C, Sudha K, Sathishkumar G, Sivaramakrishnan S (2015). Antibacterial and cytotoxic potential of silver nanoparticles synthesized using latex of *Calotropis gigantea* L. Spectrochim Acta A Mol Biomol Spectrosc.

[151] Sreekanth TV, Ravikumar S, Eom IY (2014). Green synthesized silver nanoparticles using *Nelumbo nucifera* root extract for efficient protein binding, antioxidant and cytotoxicity activities. J Photochem Photobiol B.

[152] Joseph S, Mathew B (2014). Microwave assisted facile green synthesis of silver and gold nanocatalysts using the leaf extract of *Aerva lanata*. Spectrochim Acta A Mol Biomol Spectrosc.

[153] Zuas O, Hamim N, Sampora Y (2014). Bio-synthesis of silver nanoparticles using water extract of *Myrmecodia pendan* (sarang semut plant). Mater Lett.

[154] Yousefzadi M, Rahimi Z, Ghafori V (2014). The green synthesis, characterization and antimicrobial activities of silver nanoparticles synthesized from green alga *Enteromorpha flexuosa* (wulfen). J Agardh Mater Lett.

[155] Shankar S, Jaiswal L, Aparna RSL, Prasad RGSV (2014). Synthesis, characterization, in vitro biocompatibility, and antimicrobial activity of gold, silver and gold silver alloy nanoparticles prepared from *Lansium domesticum* fruit peel extract. Mater Lett.

[156] Ghaffari-Moghaddam M, Hadi-Dabanlou R (2014). Plant mediated green synthesis and antibacterial activity of silver nanoparticles using *Crataegus douglasii* fruit extract. J Indust & Eng Chem.

[157] Shetty P, Supraja N, Garud M, Prasad TNVKV (2014). Synthesis, characterization and antimicrobial activity of *Alstonia scholaris* bark-extract-mediated silver nanoparticles. J Nanostruct Chem.

[158] Maiti S, Krishnan D, Barman G, Ghosh SK, Laha JK (2014). Antimicrobial activities of silver nanoparticles synthesized from *Lycopersicon esculentum* extract. J Analy Sci & Tech.

[159] Banerjee P, Satapathy M, Mukhopahayay A, Das P (2014). Leaf extract mediated green synthesis of silver nanoparticles from widely available Indian plants: synthesis, characterization, antimicrobial property and toxicity analysis. Biores & Bioproces.

[160] Roopan SM, Madhumitha G, Abdul Rahuman A, Kamaraj C, Bharathi A, Surendra TV (2013). Low-cost and eco-friendly phyto-synthesis of silver nanoparticles using *Cocos nucifera* coir extract and its larvicidal activity. Ind Crop Prod.

[161] Suman TY, Radhika Rajasree SR, Kanchana A, Elizabeth SB (2013). Biosynthesis, characterization and cytotoxic effect of plant mediated silver nanoparticles using *Morinda citrifolia* root extract. Colloids Surf B: Biointerfaces.

[162] ​Das S, Das J, Samadder A, Bhattacharyya SS, Das D, Khuda-Bukhsh AR (2013). Biosynthesized silver nanoparticles by ethanolic extracts of *Phytolacca decandra*, *Gelsemium sempervirens, Hydrastis canadensis* and *Thuja occidentalis* induce differential cytotoxicity through G2/M arrest in A375 cells. Colloids Surf B Biointerfaces 101:325-36. 10.1016/j.colsurfb.2012.07.008 23010037

